# Applying microscopic analytic techniques for failure analysis in electronic assemblies

**DOI:** 10.1186/s42649-019-0009-1

**Published:** 2019-08-13

**Authors:** Otto Grosshardt, Boldizsár Árpád Nagy, Anette Laetsch

**Affiliations:** 1 SC ZES Zollner Electronic SRL, 10 Parc Industrial Sud str, RO-440247 Satu-Mare, Romania; 2 Zollner Elektronik AG, 1 Manfred-Zollner Str. 1, 93499 Zandt, Germany

**Keywords:** Failure analysis, Printed circuit boards, Cross-section, Optical microscopy, SEM-EDS

## Abstract

The present paper gives an overview of surface failures, internal nonconformities and solders joint failures detected by microscopic analysis of electronic assemblies. Optical microscopy (stereomicroscopy) and Fourier-Transform-Infrared (FTIR) microscopy is used for documentation and failure localization on electronic samples surface. For internal observable conditions a metallographic cross-section analysis of the sample is required. The aim of this work is to present some internal and external observable nonconformities which frequently appear in electronic assemblies. In order to detect these nonconformities, optical microscopy, cross section analysis, FTIR-microscopy and scanning electron microscopy with energy dispersive spectrometry (SEM-EDS) were used as analytical techniques.

## Introduction

The electronic industry has shown a clear trend to miniaturization with increasing functionality. Modern printed circuit assemblies consist of a very complex labyrinth of interconnected devices, comprising many hundreds of components and thousands of individual signals being routed through a network of metal, plastic and dielectric material. To ensure a robust product, the reliability of the connections between individual components and the printed circuit board (PCB) has to be very high. Therefore, the failure analysis of electronic assemblies is a very important aspect to assure the production of high quality products. Failures on electronic assemblies can be located on: failure in the printed circuit board, defects in components and failure in the solder connection.

To assure the quality of the electronic devices, there are many analytical investigation methods such as X-Ray analysis, micro-ohm-measurements or microscopy analysis. The optical microscopic investigation is a powerful tool for the evaluation of electronic assemblies and is the most frequently applied analysis technique (Balogh et al. 2006; Castello et al. 2006; Reiter et al. 2002). It can be used to detect nonconformities on electronic assemblies and for the documentation of the samples as received (Balogh et al. 2008a, b; Nagynemedi et al. 2009; Smith 2007; Zhao and Fu 2015). After documentation, the physical appearance of the defect has to be evaluated. After these two steps of nondestructive characterization, the further way of analysis has to be established with supplementary investigation methods. In the most cases, these are destructive analysis methods, e.g. the cross-section analysis (microsectioning). Nonconformities on printed circuit boards can be divided into two main groups: external observable conditions, which can be seen and evaluated from the exterior surface of the board and internal observable conditions that requires microsectioning of the specimen (IPC-A-600 Revision J 2016).

Since electronic devices becomes smaller and smaller they are also becoming increasingly vulnerable to all kinds of contaminations. Especially the identification of contaminations which are often at microscopical scale is of particular interest as they can lead to malfunction of the electronic device. Knowledge about the chemical composition will in most cases reveals the origin of the contamination and therefore allows effective troubleshooting. FTIR-microscopy is an attractive tool for the analysis of very small structures down to the micrometer range and is capable of identifying not only organic but also inorganic components.

For internal observable conditions, a metallographic cross-section analysis of the components is required. The preparation of the samples has to take account of the individual characteristics of these materials. Electronic and microelectronic devices are made of different materials such as glass, ceramics, metals and polymers. They range from very hard materials, such as Al_2_O_3_ ceramic, up to very soft materials, e.g. tin-lead-solder, and differ in chemical composition, in crystallographic structure and in microstructure. For this reason, each individual step of the preparation should be made with maximum attention (Reiter et al. 2002):

- Cutting of samples: cracking of glass and ceramic material in components can occur.
- Mounting: mechanical deformation is possible.
- Grinding: Fracture of brittle materials (ceramic) can occur.
- Polishing: Smearing of soft materials, silicon carbide or diamond particles can remain in the cross-section.

The metallographic prepared samples (solder joints) typically require inspection using the higher magnifications available with a metallographic microscope (Castello et al. 2006).

In order to provide useful information of the surface morphology of the specimen, to analyze fracture surfaces and to establish the root cause of a nonconformity, also the scanning electron microscopy (SEM) can be used as a powerful investigation method. This allows a higher achievable magnification and depth of field than an optical microscope. Also, the SEM permits elemental analysis (Energy Dispersive X-Ray Spectroscopy-EDS), which can provide rapid qualitative analysis of elemental composition.

Taken in consideration these valuable methods, the aim of this work is to present investigations of some internal and external observable nonconformities founds on the surface of printed circuit boards, assembled printed circuit boards (PCBA) and assembled flexible boards (FBA).

In order to detect these nonconformities, optical microscopy, cross section analysis, FTIR-microscopy and SEM-EDS were used as analytical techniques. This paper will present common microscopy analytic techniques to investigate solder joint failures.

## Materials and methods

In order to identify the external observable nonconformities, a stereo microscope Olympus SZX16 was used rated between 7 and 115x magnification. At the metallographic preparation of the microsections, the identified PCBA samples were sectioned with a Secotom-1 cut-off machine (Struers, Romania). During the sectioning, the mechanical stress of the samples should be avoided. Further, the samples were washed in ethanol using an ultrasonic bath and dried for at least 20 min at 70 °C in an oven. The sample was fixed with a fixation clip and put into a mounting cup. The resin-mixture (acrylic resin ClaroCit, Struers, Romania) was filled into the mounting cup and cured for at least 20 min under a pressure of 2 bars. After hardening, the obtained microsections samples were grinded and polished with a Tegramin-30 grinding machine (Struers, Romania) using abrasive SiC-papers (Silicon Carbide Grinding discs). The preparation conditions can be seen in Tables 1 and 2. After polishing, the microsections were evaluated with an Olympus BX61 metallographic microscope at a magnification factor between 50 to 1000x.

**Table 1:** Preparation parameters for the grinding of the cross-sections

Step	1	2	3	4
Abrasive	SiC 320	SiC 1200	SiC 2000	SiC 4000
Suspension	Water	Water	Water	Water
Rotations per minute	300	300	300	300
Force (N)/specimen	20	20	20	20
Time (min)	*	*	*	*

* Depending on sample material and its grinding behavior

**Table 2:** Preparation parameters for the polishing of the cross-sections

Step	5	6	7
Abrasive	MD-Mol	MD-Nap	MD-Chem
Suspension	Diamond 3 μm	Diamond 1 μm	OP-S
Rotations per minute	150	150	150
Force (N)/specimen	20	20	20
Time (min)	2	1	0,5
Rotation direction	Co-rotation	Co-rotation	Co-rotation
Lubricant	DP-Lubricant red	DP-Lubricant red	DP-Lubricant blue

In this study, FTIR-microscopy data from the PCBA surface were collected with a Lumos FTIR-microscop (Bruker Optics, Germany).

For SEM/EDS measurements a Hitachi SU3500 (Japan) was used. The elemental composition of the failure was performed using EDS analyzer Bruker XFlash 6160 instrument.

## Results and discussion

### Failures on PCBA

The inspection by optical microscopy is used, at first, for sample documentation and failure localization. Typical failures includes: poor solder joints, cracks in components or in solder joints, solder balls, contaminations or mistakes in the marking. These failures are then investigated more closely using other microscopic techniques (cross-section analysis) to determine the exact failure and their root causes (Balogh et al., 2008a).

### Examples of nonconformities

#### Uncovered area on solder mask

**Fig. 1:**
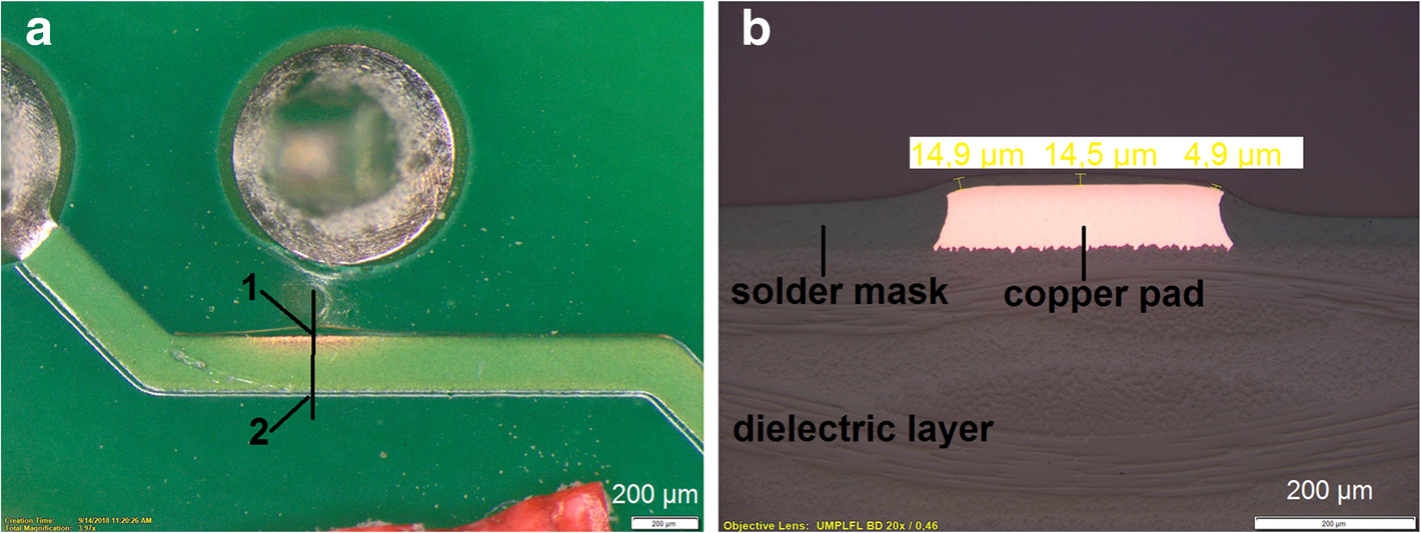
Optical microscopy evaluation of the uncovered conductor (**a**) and detailed view in the cross-section of the solder mask thickness (**b**)

The solder mask is a heat-resisting coating material applied to selected areas to prevent the deposition of solder upon those areas during subsequent soldering. The cured solder mask shall be uniform in appearance and free of any surface anomaly that would interfere with the assembly or operation of the printed board. In the Fig. 1a, the solder mask is missing from the copper conductor. The lack of metallic luster during the evaluation of the specimen indicates a too small thickness of the solder mask. The evaluation of the cross-section (Fig. 1b) shows a solder mask thickness of 15 μm in the position with acceptable solder mask thickness (position 2 in Fig. 1a) and a thickness of 4 μm in the position where the solder mask is missing (position 1 in Fig. 1a).

#### White residue on PCBA surface

**Fig. 2:**
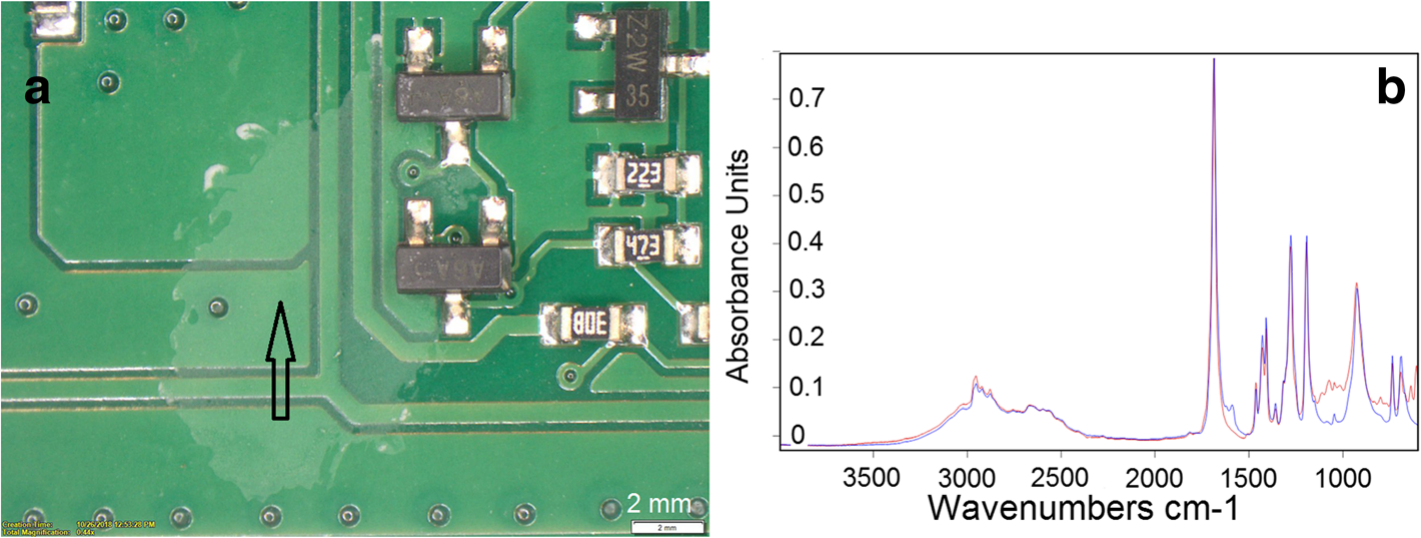
Microscopic investigation of white residues presents on the PCBA surface (**a**) and FTIR spectra analysis of the identified contamination (**b**). The recorded spectrum is marked with red line, the library spectrum with blue line

In the most cases, the appearance of the white residues indicates the presence of flux traces. Figure 2a shows a contaminated area direct on the PCB surface. In order to confirm that the observed contamination correspond to flux residues, a FTIR-microscopy analysis using the ATR-technique (attenuated total reflection) was performed directly at the contaminated area on the PCBA surface. The founded spectral data (Fig. 2b) clearly show the presence of adipic acid, the active part of the flux system. The recorded spectra (red line) fully corresponds to the library spectrum (blue line, adipic acid, Zollner own library) seen by the measured absorption bands: ca. 3000 cm^− 1^ (−O-H stretch and –C-H stretch), 1700 cm^− 1^ (−C=O stretch), 1430 cm^− 1^ (−O-H bend), 1280 cm^− 1^ (−C-O-stretch), 930 cm^− 1^ (−O-H bend). The peak near 1100 cm^− 1^ in the spectra marked with red line is attributed to solvent residues of the flux remained in the white residue. Any flux residues on electrical contact surfaces is not accepted by the standard (Huang et al. 2009; IPC-A-610 Revision G 2017).

#### Component damage

**Fig. 3:**
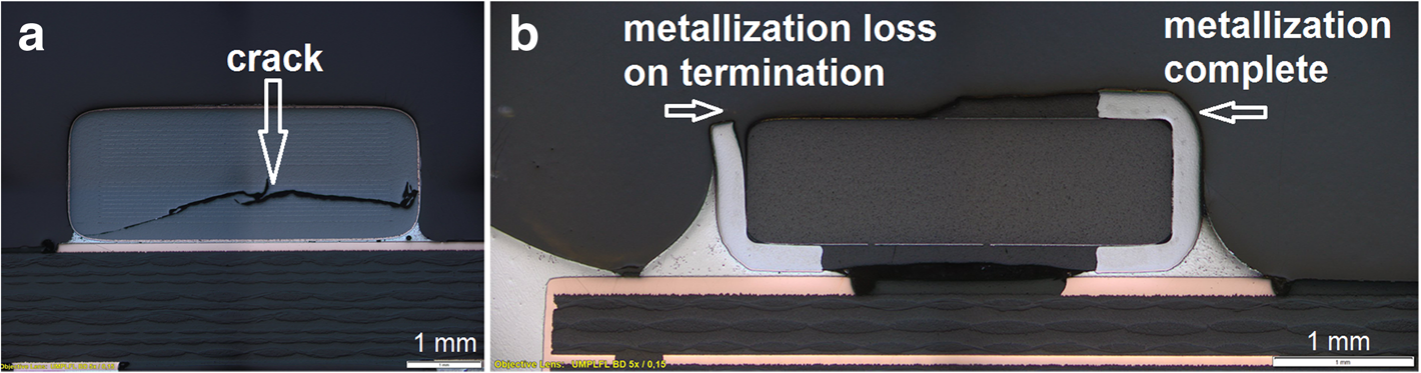
Cracked component (**a**) and metallization loss on upper termination side at a glass diode (**b**) (Figure captured using Olympus BX61 metallographic microscope at a magnification factor of 50x)

The used components should be in a perfect condition and fulfill the technical specifications. Minor deviations on surface damages can be accepted if the functionality of the component is given. Major component damages such as crack in the component body (Fig. 3a) or metallization loss on termination side (Fig. 3b) are not accepted (IPC-A-610 Revision G,^2017^).

### Failures in the solder connections

#### Examples of nonconformities

##### Voids and cavities in through hole technology (THT) solder joint

**Fig. 4:**
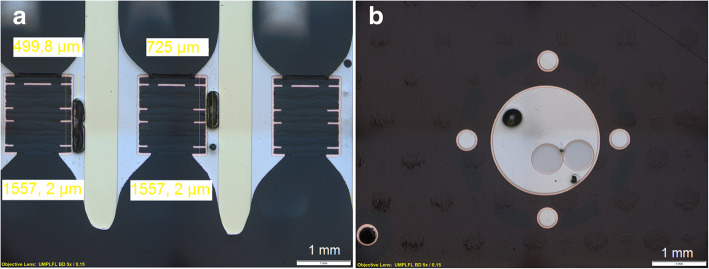
Pores and cavities in through-hole solder joint. The measured vertical fill with solder paste for the left solder joint is 499.8 μm, the height of the THT hole is 1557.2 μm. So the vertical fill with solder paste is calculated by (499.8/1557.2) × 100% = 32.1%. The void in the left solder joint is about 67,9% of the vertical height of the solder joint, in the right solder joint the void is about 53,3%. The vertical fill with solder paste is < 75% as specified in the standard, so that this solder joints are not accepted (**a**). Also in the horizontal cross-section voids can be found in the solder joint. In this case the solder joint is well formed, the void does not influence the mechanical stability of the solder connection (**b**) (Figure captured using Olympus BX61 metallographic microscope at a magnification factor of 50x)

In this example (Fig. 4a and b) voids and cavities are visible in the solder joints for the through holes components. They are due to outgassing from the organic base material contained in flux. Possible causes for the outgassing occurrence are the humidity in the base material, organic contamination on the leads, pins and holes. The large cavities increase the susceptibility for voids of solder joints and therefore, impair their mechanical robustness. These voids and cavities are not visible from outside; an inspection is not possible excepting an X-Ray analysis. Only the cross-cut analysis can detect the size of the cavities and pores. The vertical fill with solder paste is < 75% (measured value: 32,1%) as specified in the standard, so that this solder joints are not acceptable (IPC-A-610 Revision G 2017). These solder joints should undergo rework (possible reflow).

##### Copper leaching

**Fig. 5:**
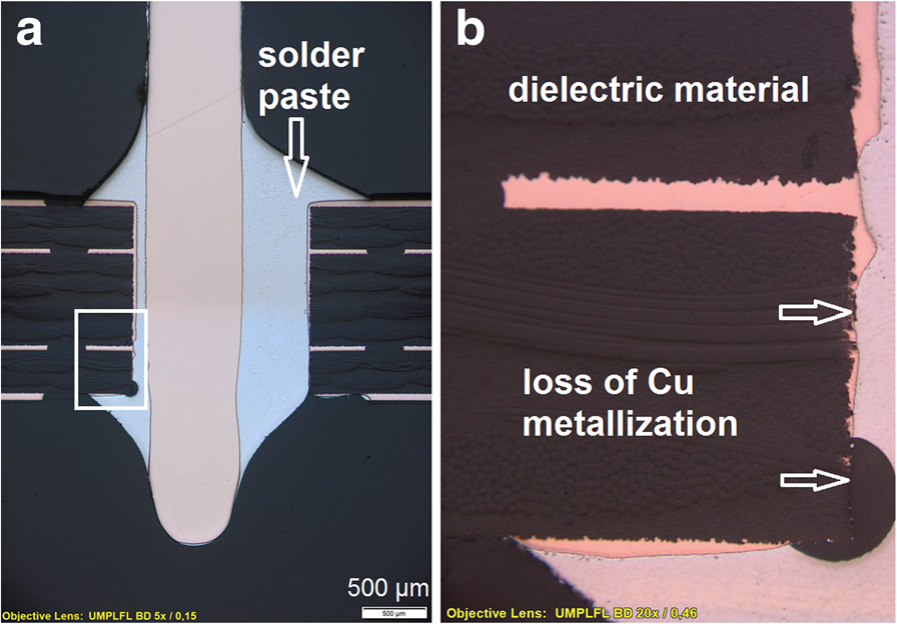
Phenomena of copper leaching (**a**). The copper wall of the plated through hole is disappeared because copper is solved in the solder paste (**b**). (Figure captured using Olympus BX61 metallographic microscope at a magnification factor of 50x and 100x)

In Fig. 5 the phenomena of copper dissolution can be observed. The copper wall of the plated through hole is disappeared because the copper is solved in the solder paste (Fig. 5 phenomena was marked with white arrows). Not only disappearance but also thinning of copper layers may induce to disconnection problems, which are not desirable from the viewpoint of the joint reliability. Possible cause can be a too high soldering temperature or a too long soldering time. The Sn-Ag-Cu alloy is reactive to metals (for example copper or iron), the dissolution or/and disappearance of copper layers is a problem on wave soldering where PCBs are dipped in molten solder paste (Izuta et al. 2007; Snugovsky et al. 2009).

##### Crack in the solder joint and black pad phenomena

**Fig. 6:**
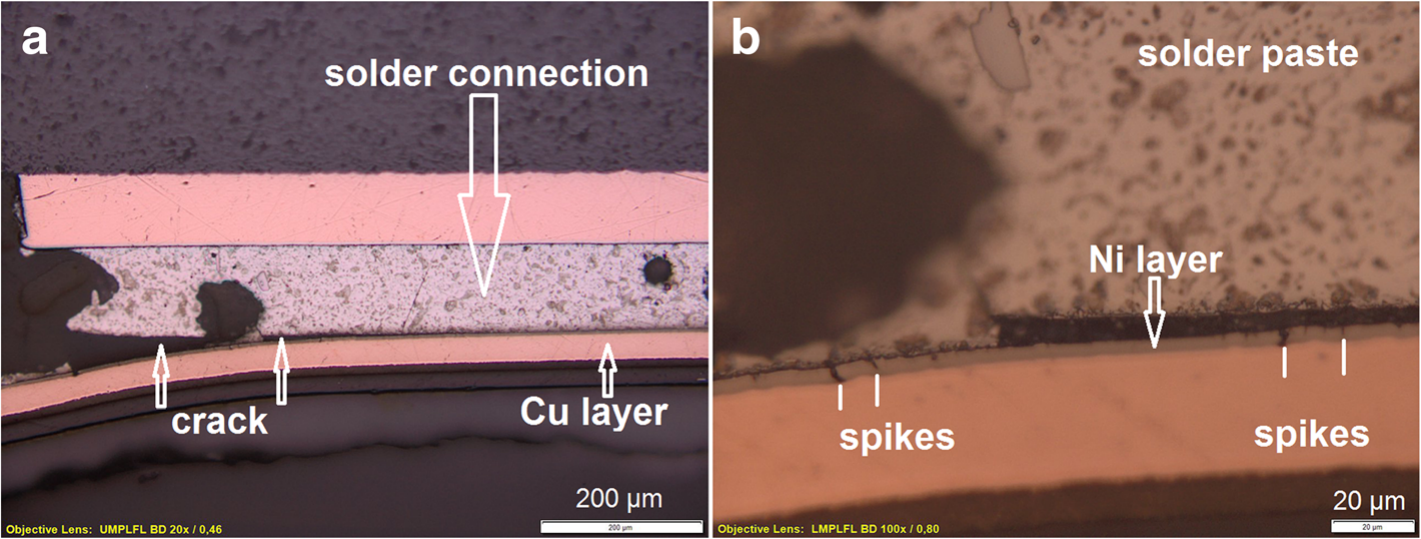
At the component a crack between the solder joint at the pad was found by microscopic analysis of the cross-section (**a**). The Black Pad phenomena: corrosion spikes are visible in the nickel layer (**b**)

At the presented component soldered on a FBA an electric malfunction was found. To determine the root-cause of the defect, a microscopic verification of the prepared cross-section was done. The microscopic examination approve the presence of a crack in the solder joint (Fig. 6a). The crack is formed between the intermetallic layer and the Nickel-layer of the pad on a PCB with ENIG surface (electroless nickel/immersion gold). Figure 6b shows a corroded Black Pad structure: corrosion spikes are visible in the nickel layer. Black Pad failures are a nickel corrosion process. The main features of the phenomenon are partial wetting by solder of certain ENIG areas, the presence of Ni surface morphology defects (mud cracks, spikes) and black spots. The Black Pad phenomena in the corroded nickel surface results in the loss of solderability and that has caused poorly formed solder joints at the interface between the solder and the nickel interface. When such weakened solder joints are subjected to mechanical stress, they can be easily fractured (Hui Lee 2011; Ramanauskas et al. 2013; Snugovsky et al. 2001; Snugovsky, 2002; Susan et al. 2004).

##### Damaged solder joint and fallen component

**Fig. 7:**
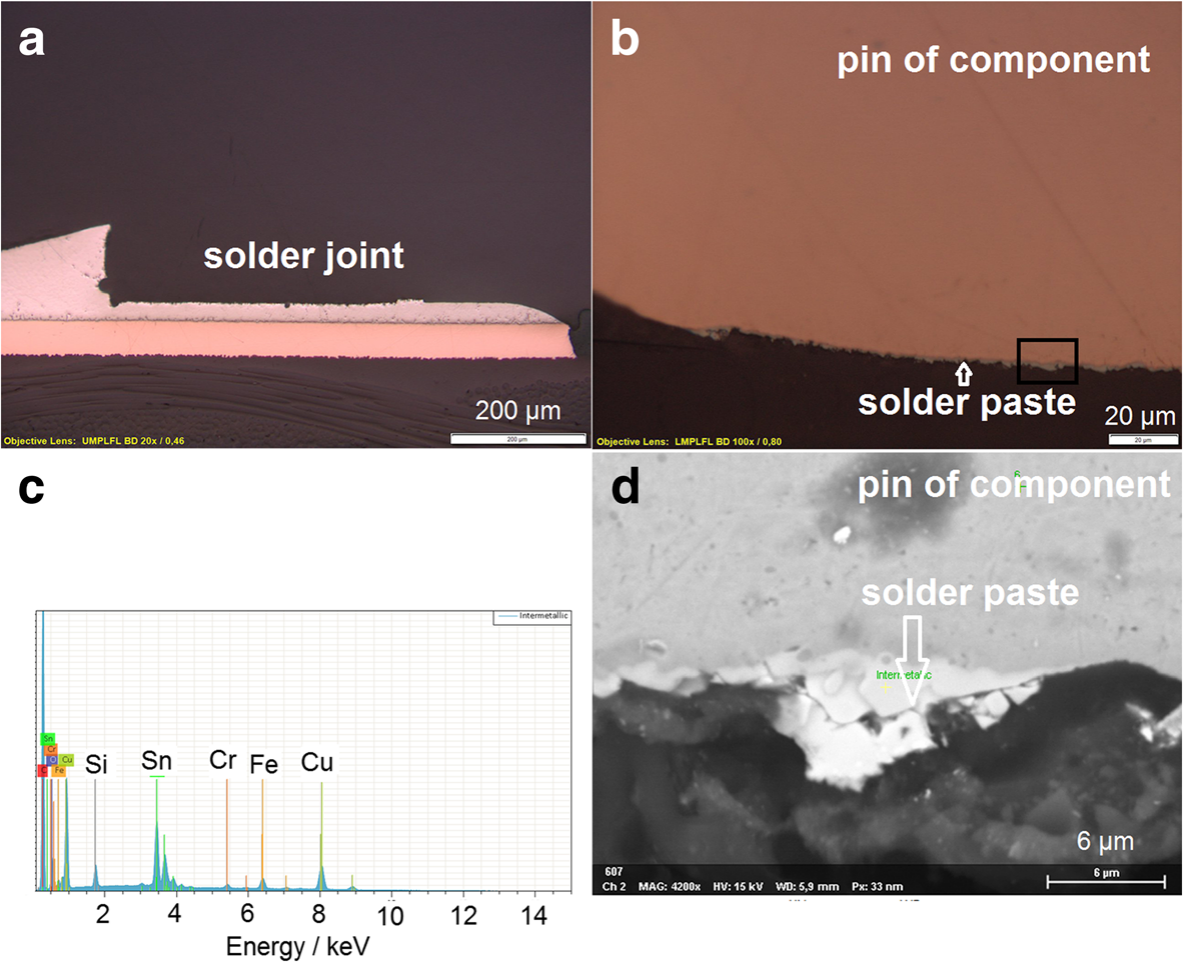
Microscopic overview of solder joint with missing component (**a**). Missing component (**b**). EDS-analysis of solder paste (**c**). SEM analysis of component pin (**d**)

In the present case a missing component from the assembled printed circuit board was observed (Fig. 7a). The component was separated from the solder joint where the original form of them can be observed. The microscopic verification of the components pin at the maximum magnification factor of the metallographic microscope (1000x) confirms the presence of solder paste (Fig. 7b). The use of a scanning electron microscope which allows a higher magnification (picture was taken at a magnification factor of 4200x) indicates the presence of solder paste on the pin (Fig. 7d). The EDS-analysis performed at this point shows the presence of tin and copper, which are the mainly elements in the composition of the solder paste and of the intermetallic layer (Fig. 7c). The evidence of tin on the components pin shows that the pin was soldered to the PCB. Probably due to a mechanical stress the strength of the solder joint became weaker and the component was separated from the PCB.

##### BGA (ball grid array) defect

**Fig. 8:**
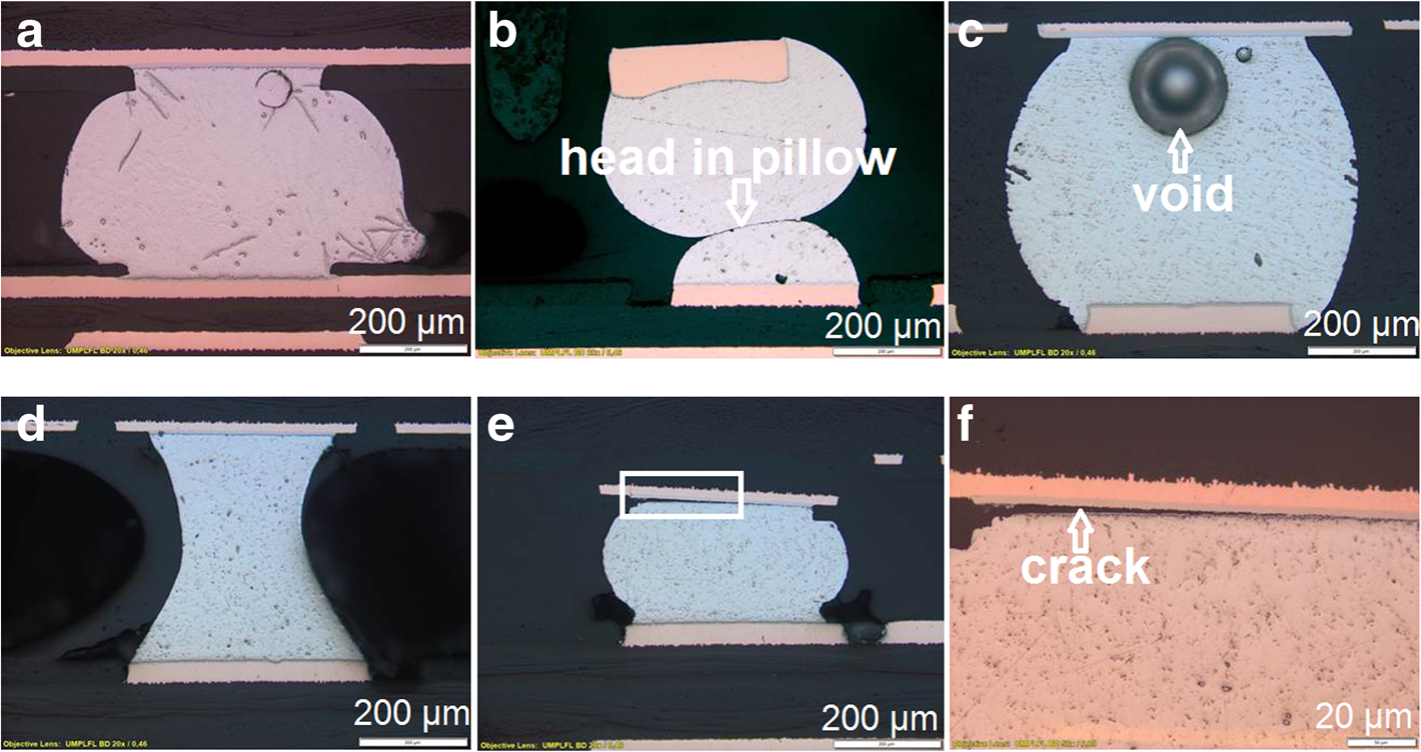
Possible soldering failures on BGA components: deformed solder ball (**a** and **d**), head in pillow (**b**), voids (**c**) and crack in solder joint (**e** and **f**)

A ball grid array (BGA) is a type of surface-mounted devices used for integrated circuits. BGAs use solder bump interconnections instead of pins. Once the package is soldered into place, it is difficult to find soldering faults. X-ray analysis, industrial computed tomography scanning and endoscopy are non-destructive analysis methods which can be used to identify the soldering faults. To find the real root cause of the soldering fault the cross-section analysis offers good possibilities to reach this goal. Figure 8 shows possible soldering failures on BGA components:

- ➡ BGA ball with macrovoid (Fig. 8c). Macrovoids are caused by volatile compounds that evolve during the soldering processes. These macrovoids generally do not affect the solder joint reliability unless they are present at interfacial regions in the solder joints where cracks typically propagate.
- ➡ Head-in-pillow (Fig. 8b) is a solder joint defect where the solder paste wets the pad, but does not fully wet the ball. For example, in the case of a ball grid array, the predeposited solder balls on the component and the solder paste applied to the circuit board may both melt, but the melted solder does not join. The cross-section through the failed joint shows a distinct boundary between the solder ball on the component and the solder paste on the circuit board. This solder joint fulfills the conditions for a connection to have electrical integrity, but is lacking sufficient mechanical strength. Due to the lack of solder joint strength, this component may fail with very little mechanical or thermal stress. The defect was probably caused by surface oxidation or poor wetting of the solder.
- ➡ Deformed solder ball (Fig. 8a, d). The dynamic warpage of the PCB and/or BGA can lead to varying shapes of solder joints. The left solder joint in the upper row (Fig. 8a) shows a convex solder joint. The warpage can lead also to solder joint being stretched into a columnar shape (figure left in the lower row Fig. 8d). Both solder joints are acceptable from a quality standpoint (IPC-7095D 2018).
- ➡ Crack in solder joint (Fig. 8e, f). A cracked or fractured solder ball is not accepted. This failure can be caused by mechanical stress, for example the BGA was not aligned parallel to the PCB surface (Champaign and Wiggins 2007; Hofmeister et al. 2008).

## Conclusion

In this study, various microscopic analytic techniques have been successfully used to characterize failure samples and nonconformities in electronic assemblies. The applied techniques, optical microscopy, FTIR-microscopy and SEM combined with EDS were used to locate and describe the failure and to reveal the root cause. The inspection of the sample by optical microscopy is used for sample documentation and failure localization. Externally observable conditions can be detected by this visual evaluation of the electronic assembly. Also the sources of contaminations such as flux can be identified by FTIR-microscopy. The achievable magnification of the optical microscopy is above 7 to 115x, so this method cannot fulfill the needs of the metallographic analysis of the cross sectioned solder joint. For this purpose, cross sectioning of the sample can be used. With this method also internally observable conditions can be characterized. For in depth analysis to understand the failure and the root cause of it, the cross sectional microscopic investigation with metallographic microscopy and SEM-EDS were successfully used. As a result a failure analysis methodology was described based on various microscopic analytic techniques which allows to reveal the root causes of electronic assembly failures.

## Data Availability

Not applicable.

## Abbreviations

BGA: Ball grid array
FBA: Assembled flexible boards
FTIR: Fourier-Transform-Infrared
PCB: Printed circuit board
PCBA: Assembled printed circuit boards
SEM-EDS: Scanning electron microscopy with energy dispersive spectrometry
THT: Through hole technology
